# The diatom *Thalassiosira gravida* under Arctic change: algal exudates and their microbial transformation under contrasting temperatures and photoperiods

**DOI:** 10.1128/aem.01429-26

**Published:** 2026-09-04

**Authors:** Frederik Bussmann, Jakob K. Giesler, Sylke Wohlrab, Oliver J. Lechtenfeld, Jan Kaesler, Tilmann Harder, Boris P. Koch

**Affiliations:** 1 Alfred-Wegener-Institut Helmholtz-Zentrum für Polar- und Meeresforschung 84597 , Bremerhaven, Germany; 2 University of Bremen 9168, Bremen, Germany; 3 Helmholtz Institute for Functional Marine Biodiversity at the University of Oldenburg (HIFMB) 577550 , Oldenburg, Germany; 4 Helmholtz Centre for Environmental Research - UFZ 28342, Leipzig, Germany; 5 University of Applied Science, Bremerhaven, Germany; University of Milano-Bicocca, Milan, Italy

**Keywords:** diatom, extracellular release, dissolved organic carbon, dissolved organic matter, bacterial transformation, *Thalassiosira gravida*, microbial carbon pump, FT-ICR-MS

## Abstract

Climate change threatens polar ecosystems through rising temperatures and changing light regimes. Phytoplankton release of dissolved organic carbon (DOC) is a major carbon-cycle component, yet it remains unclear how temperature, light, and associated bacteria jointly affect DOC and dissolved organic matter (DOM) composition. Here, we analyzed axenic and xenic cultures of the Arctic diatom *Thalassiosira gravida* at 9°C and 13.5°C under 16:8 h and 24:0 h light:dark cycles. Extracellular DOC was quantified, and molecular composition characterized by untargeted ultrahigh-resolution mass spectrometry in original culture filtrates. In axenic cultures, growth rates were 266% higher at 13.5°C 24:0 h, and cell-normalized DOC concentrations 52% lower compared to 9°C 16:8 h, suggesting a shift in carbon allocation. Xenic cultures contained 50–80% lower cell-normalized DOC concentrations than axenic cultures and converged at 11.7 ± 0.9 pmol DOC cell^−1^. Despite similar DOC concentrations in xenic cultures, the 13.5°C 24:0 h treatment showed lower chemodiversity (−8%), H/C ratios (−7%), and higher oxidation (+24%) than 9°C 16:8 h, consistent with more strongly processed DOM. At 9°C, axenic 24:0 h cultures showed higher chemodiversity (+14%) and H/C ratios (+7%) compared to 16:8 h, indicating that prolonged photoperiods altered DOM composition toward signatures associated with greater putative bioavailability. Contrasting axenic-xenic patterns suggest that bacterial presence weakened the treatment-dependent DOM responses observed in axenic cultures. Overall, temperature and photoperiods altered algal DOC quantity and quality, and its microbial transformation, with implications for carbon cycling.

**IMPORTANCE:**

The Arctic Ocean is warming rapidly, and changing sea-ice conditions alter light availability. Phytoplankton release dissolved organic carbon (DOC), which can be transformed by associated bacteria, thus playing an essential role in the marine carbon cycle. Still, the fate of such DOC transformations remains difficult to predict in the scope of future climate change scenarios. Using axenic and xenic cultures of the Arctic diatom *Thalassiosira gravida* as a model, our findings emphasize that algal extracellular release and its microbial transformation must be evaluated not only in the framework of bulk DOC dynamics but also with respect to molecular diversity and composition. Therefore, physiological responses of Arctic phytoplankton to higher temperatures and prolonged photoperiods may affect algal carbon exudation as well as the diversity and composition of microbially processed DOM in a warming Arctic Ocean.

## INTRODUCTION

Oceans contribute ~50% to global primary production by unicellular phytoplankton (1). Thus, phytoplankton fuels the pool of dissolved organic matter (DOM) (2, 3). Key processes of DOM release include extracellular release (4, 5), senescence of phytoplankton blooms (6), for example, via programmed cell death (7), viral lysis (8, 9), or sloppy feeding or egestion by metazoan grazers (10–13). Most freshly released compounds are highly bioavailable and rapidly consumed (2), i.e., part of the labile dissolved organic carbon pool. Heterotrophic prokaryotes can metabolize DOC, diversify DOM (14), and, in combination with photochemical transformations, contribute to recalcitrant DOC (RDOC), characterized by compounds with residence times of millennia. Such transformations are consistent with the concepts of the microbial loop (2) and the microbial carbon pump (MCP) (15). Apart from the utilization of algal DOC, prokaryotes themselves contribute to marine DOC via chemical transformation, excretion, or viral lysis (16). Together, the carbon fixation by phytoplankton and bacterial processing fundamentally act as a long-term sink for atmospheric CO_2_ (17–20).

Marine ecosystems are threatened by climate change (21), especially in Arctic regions (22, 23), due to positive feedback processes known as Arctic amplification (24, 25). Microbial processes in polar ecosystems are particularly sensitive to small environmental changes because of their specialized adaptations and fragile habitats (26, 27), which leads to extensively altered phytoplankton and bacterial productivity dynamics (28–31). Arctic sea surface temperatures have markedly risen since the 1890s (32). During the transition from spring to summer, pelagic phytoplankton is exposed to increased irradiation and prolonged photoperiods caused by flash snow melting and ice breakup (33). Light conditions in seasonally sea-ice covered areas are particularly affected by climate change (34). As a result of more open, less saline, and warmer surface waters with lower ice coverage, stratification is increasing (35), confining phytoplankton near the surface. Consequently, higher seawater temperatures and more frequent exposures to prolonged light periods are expected (36, 37). Since the Arctic Ocean is generally characterized by extreme light regimes with constant light conditions during polar day and constant darkness during polar night, phototrophic carbon acquisition and primary production are dominated by periodic changes of light availability (38, 39). Thus, rising ocean temperatures may co-occur with prolonged periods of light availability, but its combined effects on polar phytoplankton are poorly understood (26). While these interactive effects on phytoplankton growth received some attention (40, 41), less is known about how these factors impact the composition of phytoplankton DOM. Investigating the multifactorial effects of environmental change on the physiology of organisms as well as overall ecology has been gaining research attention, emphasizing the importance of the interacting effects of global change (42, 43).

While (eco-)physiology and growth of phytoplankton are known to be affected by environmental changes (44–46), comparatively little is known about environmental effects on the extracellular release of DOM (47). Two models describing extracellular release by phytoplankton have been suggested: the overflow model and the passive diffusion model (48). According to the overflow model, DOM is actively released due to excess carbon that is fixed by photosynthesis but cannot be used for biomass growth or used for anabolism. This concept is derived from the observation that excess photosynthate is released under nutrient limitation (49, 50). The passive diffusion model describes the unintended maintenance of a DOC concentration gradient across the cell membrane which provides low-molecular weight (LMW) compounds for consumption by heterotrophs (51). There is evidence for both models, and they can occur simultaneously depending on environmental conditions and community structure. Concomitantly, the combined effect of varying temperature and light regimes may shift the rate and molecular composition of extracellular release, and thereby the bioavailability of DOM for heterotrophic processing. We therefore used a multifactorial design combining temperature, photoperiod, and microbial presence to assess their relative contributions.

Specifically, we analyzed the extracellular release of axenic and xenic cultures of *Thalassiosira gravida*, a dominant diatom in the Arctic Ocean (52). We aimed to understand how extracellular release and metabolization of DOC are quantitatively and qualitatively affected by temperatures below and above this species’ thermal optimum under spring and summer light regimes, and in the presence/absence of bacteria, e.g., with and without bacterial utilization of extracellular DOC. Based on a novel LC-FT-ICR MS method (53), which allowed direct analysis without any preceding purification steps (e.g., solid-phase extraction; SPE), we chemically characterized extracellularly released DOM in original filtrates from the diatom cultures during the exponential growth phase. To relate molecular-level observations to experimental conditions, we calculated the hydrogen-to-carbon (H/C) and the oxygen-to-carbon (O/C) ratios, the nominal oxidation state of carbon (NOSC), and indices describing the unsaturation (double-bond equivalents; DBE) and aromaticity (aromaticity index; AI). Since these metrics serve as indicators for the oxidation state and structural features of DOM, we applied them to assess whether 13.5°C 24:0 h conditions shift extracellular DOM toward more oxidized and/or saturated/aromatic compounds and whether microbial processing in xenic cultures removes labile fractions of DOM.

We hypothesized the following.

- Exposure of axenic *T. gravida* cultures to 13.5°C 24:0 h conditions results in lower specific growth rates (according to reference 54) compared to those at 9°C 16:8 h. We further aim to investigate treatment-dependent extracellular DOC accumulation.
- In axenic cultures, warming and/or prolonged photoperiods alter the net extracellular DOM signature compared to 9°C 16:8 h conditions, as reflected by changes in chemodiversity and molecular indicators related to putative bioavailability.
- Under 13.5°C 24:0 h conditions, xenic cultures show lower extracellular DOC accumulation and a stronger net processed extracellular DOM signature compared to 9°C 16:8 h conditions (due to putative enhanced metabolic rates).
- Due to microbial turnover, bacterial presence reduces the treatment-dependent extracellular DOM response patterns observed in axenic cultures of *T. gravida*.

## MATERIALS AND METHODS

All glassware was combusted for 12 h at 500°C prior to usage.

### Cultivation

A xenic polar strain of *Thalassiosira gravida* was obtained from the Norwegian Culture Collection of Algae (NORCCA strain ID UIO 478) and cultivated at 4°C with 50 µmol photons m^−2^ s^−1^ and a photoperiod of 16:8 h. The culture was grown in enriched sterile-filtered artificial sea water (ESAW) (55) and kept in the exponential growth phase by weekly semi-continuous dilution (1:100) with ESAW medium to ensure that culture volume and nutrient concentration remain constant (56). To reduce potential contamination, sodium chloride, sodium sulfate, and potassium chloride were combusted prior to ESAW preparation.

To acclimate the cultures to the experimental conditions, 500 mL stock cultures of xenic and axenic *T. gravida* were inoculated at 5,000 cells mL^−1^ in combusted 500 mL Erlenmeyer flasks (sealed with combusted aluminum caps) and acclimated to their respective experimental conditions, consisting of temperature (9°C and 13.5°C) in combination with different photoperiods (24:0 h and 16:8 h), at a light intensity of 55 µmol photons m^−2^ s^−1^. Here, “acclimation” refers to short-term physiological adjustment of the diatom cultures to the experimental temperature and light regime. Based on previous work, we assumed that photosynthetic acclimation was achieved within this duration (57). During the acclimation phase, stock cultures were semi-continuously diluted daily, i.e., through regular substitution of old culture medium with freshly prepared modified ESAW medium to maintain them in exponential growth and facilitate stable nutrient conditions. Axenicity was initially achieved according to reference 58. To ensure that no traces of antibiotics were present in the diatom cultures, axenification was conducted at least 5 months prior to the start of the experiment, and cultures were washed with ESAW. In axenic stock cultures, the absence of bacterial contaminants was controlled every 5 to 7 days by epifluorescence microscopy at 400× magnification using SYBR Green as a staining agent. After 7 days of acclimation, 200 mL experimental units were inoculated from the stock cultures at an initial cell density of 1,500 cells mL^−1^ in combusted 250 mL Erlenmeyer flasks (sealed with combusted aluminum caps) with five replicate cultures per treatment. Twice per day, the Erlenmeyer flasks were carefully homogenized and their locations randomized on LED light tables (CLF Plant Climatics) within their respective climate cabinets. To minimize contamination, one replicate per treatment was opened for daily cell density monitoring, while the remaining experimental units were kept sealed until sampling to avoid contamination.

### Sampling

Cell density counts were performed daily to determine the harvest time point using one replicate per treatment to monitor growth and viability and determine the time point for sampling. For this purpose, three 0.5 mL subsamples were sampled daily from the respective replicate and counted using an inverted light microscope (Zeiss Axiovert 10C) (40).

Since the extracellular release depends on growth phase and species (59, 60), cultures were harvested at a cell density of ~15,000 cells mL^−1^ to ensure that cultures were still at exponential growth at the time point of sampling. To avoid putative errors due to the circadian rhythm, the harvest was conducted between 1 and 3 PM. Thirty milliliters of spent culture medium was filtered through a 0.2 µm polycarbonate filter (Whatman) that was cleaned by soaking in 10% hydrochloric acid (HCl, Merck Suprapur) for at least 12 h and subsequently rinsed with ultrapure water (Merck Millipore Milli-Q). Glass filtration units (Sartorius) were used, and a vacuum was applied (200 mbar). Two milliliters of the filtrates was pipetted into 2 mL glass vials (Agilent) for LC-FT-ICR-MS analysis; the remainder was stored in acid-rinsed (HCl, Merck Suprapur) high-density polyethylene (HDPE) flasks. All samples were stored at −20°C until further analysis. The following blanks were sampled: (i) cultivation medium without organisms, (ii) medium filtered according to sampling procedure, (iii) ultrapure water filtered according to sampling procedure, (iv) ultrapure water processed as samples cultivated in a 9°C incubator, and (v) ultrapure water processed as samples cultivated in a 13.5°C incubator.

### Diatom growth rates

Specific growth rates (*µ*) were calculated as follows (Eq. 1):


(Eq. 1) $$\mu =\frac{\ln(N_{end})-\ln(N_{0})}{\Delta t}$$

where *N*_end_ and *N*_0_ represent the cell density (cells × mL^−1^) at the end of the cultivation and the start of the inoculation, respectively. Δ*t* refers to the time span (days) between the assessment of *N*_end_ and *N*_0_.

### Bacterial community analysis

Bacterial community structure was assessed using 16S rRNA gene amplicon sequencing data (61). In brief, 20 mL culture was prefiltered through a 10 µm nylon filter to retain diatoms, and the filtrate was collected on a 0.22 µm polycarbonate filter membrane. The filter was stored in SL1 lysis solution and frozen at −20°C until further processing. DNA extraction was conducted following the manufacturer’s protocol (Nucleo Spin Soil extraction kit, Macherey-Nagel GmbH, Düren, Germany). Following DNA extraction, sequencing of the V4 region of the 16S rRNA gene was performed following the Illumina 16S Metagenomic Sequencing Library Preparation protocol (protocol #15044223 B; Illumina, San Diego, CA, USA). PCR reaction was conducted using the primers MS_v4_515F_N: 5′-TCGTCGGCAGCGTCAGATGTGTATAAGAGACAG + GTGCCAGCMGCCGCGGTAA-3′ and: MS_v4_806R_1: 5′-GTCGTCGGCAGCGTCAGATGTGTATAAGAGACAG + GGACTACHVGGGTWTCTAAT-3′ (62). Bioinformatic data processing was conducted as described in detail in reference 63.

### DOC analysis and calculation of net cell-specific DOC accumulation rate

Quantification of DOC was determined by high-temperature catalytic oxidation with a Shimadzu TOC analyzer (VCPN-TOC, Shimadzu) according to reference 64. Prior to measurement, 3.25 mL of sample filtrate were diluted with 3.25 mL of ultrapure water. Ultrapure water blanks were below the limit of quantification of 19.1 µmol L^−1^. To avoid biases due to different cell densities, DOC concentrations were normalized to the respective cell densities of *T. gravida*. Additionally, to account for differences in cultivation time and cell abundance, a net cell-specific DOC accumulation rate was estimated for each sample according to Eq. 2.


(Eq. 2) $$\frac{\left(\frac{c{\left(DOC\right)}_{t1}-c{\left(DOC\right)}_{t0}}{\Delta t}\right)}{\left(\frac{N_{0}+N_{t}}{2}\right)}$$

where DOC_*t*0_ was approximated by the original DOC concentration of the sterile medium blank (272.8 µmol × L^−1^), DOC_*t*1_ represents the original DOC concentration at the time of harvest, and Δ*t* represents the incubation time. Mean cell abundance was approximated by the arithmetic mean of the cell count at the time of inoculation (*N*_0_) and harvest (*N*_*t*_).

### LC-FT-ICR-MS analysis

Two milliliters of the sample was filtered through a 0.2 µm regenerated cellulose (RC) membrane syringe filter (Sartorius) after defrosting. Measurements were conducted as described in (53). In brief, 100 µL of the samples were directly injected onto a polar end-capped C-18 reversed-phase column (ACQUITY HSS T3, 1.8 μm, 100 Å, 150 × 3 mm, Waters, Milford, MA, USA) on an ultrahigh-pressure chromatography system (UHPLC; UltiMate 3000RS, Thermo Fisher Scientific, Waltham, MA, USA). Ultrapure water (pH 3) and methanol (LC-MS-grade, Biosolve, Valkenswaard, Netherlands) were used as mobile phases. Separation of salt was achieved by an extended isocratic 100% aqueous step followed by an increase in methanol until 100% (14 min). A post-column counter-gradient was added via T-piece. A 2-way-6-port valve diverted early eluent to waste (switch to ESI at 10.5 min) before measurement (Bruker solariX XR FT-ICR-MS). MS data were recorded only during the DOM elution window (12.5 min–26.5 min) to (i) avoid matrix effects caused by the salt and (ii) stop before 100% methanol wash phase, where spectra are dominated by the solvent. Ion accumulation time (IAT) was adjusted to 600 ms due to high DOC concentrations of the samples. LC-MS scans were averaged into 1 min retention-time segments for formula assignment. The assignment was conducted using UltraMassExplorer (UME) (65). Filter settings were applied according to reference 66.

The total ion chromatogram (TIC) describes the sum of intensity of all detected ions in a mass spectrum and serves as a measure for overall ion abundance. In this study, it was used to compare samples and to ensure that differences in the DOM composition were not primarily driven by signal variability. One outlier was removed due to an atypical TIC profile (axenic 13.5°C 16:8 h data set).

From the assigned molecular formulas, the weighted averages (wa) of the H/C ratio, the O/C ratio, NOSC, double-bond equivalents (DBE), and the AI were calculated. DBE and AI were calculated according to (67, 68). DBE represents the sum of unsaturations plus rings in a molecule. Dividing DBE by the number of C atoms gives an unsaturation density (69). The AI builds on DBE/C ratio (70) and can indicate the presence of aromatic or condensed-aromatic substructures in a molecule. AI comes with the assumption that triple bonds, cumulated double bonds, and small and bridged ring systems are negligible in DOM and that the presence of heteroatoms can contribute to non-aromatic unsaturations (67, 71).

The ratio of the elements C, H, N, O, and S can be used to estimate the energetic potential of compounds (72). Based on the element ratios, NOSC calculated and represents the average oxidation state of carbon atoms in a molecule. NOSC was shown to be correlated with the standard molal Gibbs energies of the oxidation half-reactions of organic compounds (72). In a relative sense (i.e., among treatments), negative values indicate reduced carbon, while positive values indicate more oxidized carbon.

### Dissolved macronutrients

Samples (1.5 mL) were diluted with artificial seawater (13.5 mL) and analyzed using a Seal AA500 auto-analyzer. Analyses followed (73), with modifications according to the manufacturer’s methods (Seal, Alliance). Measurements were validated against the certified reference material Jamstec 7602a and 7603a (*n* = 4) provided by the National Metrology Institute of Japan.

### Statistical analyses, assessment of chemodiversity, and visualization

All calculations, statistical analyses, and graphs were made using R Studio (version 4.5.1; https://www.r-project.org/). Treatments effects were assessed separately for axenic and xenic cultures using a Kruskal–Wallis test. After that, a Dunn’s *post hoc* test (*α* = 0.05) was applied for specific growth rates, normalized DOC concentrations, net cell-specific DOC accumulation rates, richness, Shannon diversity indices, evenness, H/C ratios, O/C ratios, and NOSC using the packages “rstatix” (version 0.7.3) (74). *P* values were adjusted for multiple testing (5% false discovery rate) (75). Compact letter displays (CLD) were created using the package “rcompanion” (version 2.5.0) (76). In addition, to compare axenic and xenic cultures within their respective treatment, a Mann-Whitney *U* test (77) was applied (R package “coin”) (71). Exact *P* values were calculated, and effect sizes were quantified using Cliff’s delta (78).

Chemodiversity was assessed by calculating the number of molecular formulas per sample (richness; S) and the Shannon diversity index (H) (79), as follows (Eq. 3):


(Eq. 3) $$H=\;-\sum_{i=1}^{S}\frac{n_{i}}{N}\times ln\left(\frac{n_{i}}{N}\right)$$

where *n*_*i*_ is the intensity of a molecular formula *i* and *N* the total intensity of the sample. Pielou’s evenness (80) equals the quotient of the Shannon index and natural logarithm of richness. Peak intensities summed over retention times were used for calculations. Since signal intensities do not directly translate to concentrations, these indices represent signal-based diversity derived from ion abundances (81). All calculations were performed using the “vegan” package (version 2.7.1) (82).

Boxplots were created using “ggplot2” (83). Data are presented as Tukey boxplots. For the van Krevelen diagram, “tidyverse” packages were applied (version 2.0.0) (84).

## RESULTS

Based on pre-determined thermal performance curves of *T. gravid*a (54), two temperatures were selected. The lower experimental temperature (9°C) falls within the high-performance range of *T. gravida*, while the higher experimental temperature (13.5°C) exceeds the thermal optimum and is expected to provoke physiological stress. These experimental temperatures were combined with a 16:8 h light:dark cycle and continuous light (24:0 h light:dark) to determine the combined effects of temperature and photoperiod to assess quantity and quality of extracellular DOM in axenic and xenic cultures.

Lowercase letters above each box in the figures indicate the significance levels from the Dunn’s *post hoc* test comparing the four applied treatments within axenic and xenic cultures. For the comparison between axenic and xenic cultures for each treatment, the reported *P* values refer to exact two-sided values from the Mann-Whitney *U* test (Table S4).

### Diatom growth rates

**Fig 1:**
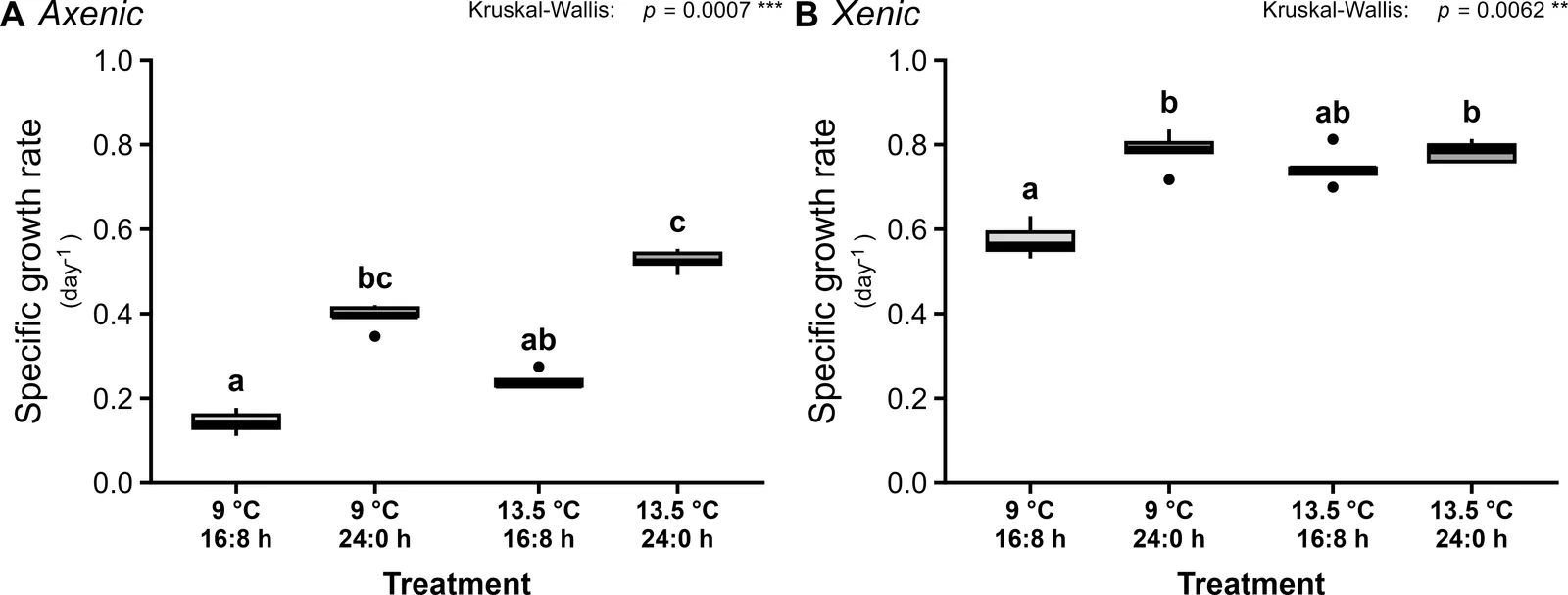
Box plots of specific growth rates of axenic (**A**) and xenic (**B**) cultures of *T. gravida* under different temperatures (9°C, 13.5°C) and photoperiods (16:8 h, 24:0 h). Each treatment consisted of five replicates with the exception of axenic cultures at 13.5°C and 16:8 h (*n* = 4). Treatments with different lowercase letters differed significantly (*P* < 0.05).

Prolonged photoperiods (24:0 h) resulted in significantly higher specific growth rates compared to the corresponding 16:8 h photoperiodic conditions at the same temperature (*P* < 0.05, Fig. 1), except for the xenic treatments at 13.5°C (Fig. 1B). In axenic cultures, specific growth rates under constant light were 175% higher at 9°C and 118% higher at 13.5°C compared to the corresponding 16:8 h photoperiod (*P* < 0.05, Fig. 1A). Moreover, specific growth rates of the 13.5°C 24:0 h in axenic cultures were 266% higher than those at 9°C 16:8 h (*P* < 0.05, Fig. 1A).

In xenic cultures, the 9°C 24:0 h and 13.5°C 24:0 h treatments showed significantly higher specific growth rates than the 9°C 16:8 h, both by 37% (*P* < 0.05, Fig. 1B).

### Community composition of free-living bacteria in xenic cultures

The composition of free-living bacterial communities based on 16S rRNA gene amplicon data were analyzed to provide taxonomic context for the xenic cultures. Across all xenic treatments, bacterial communities were dominated by *Colwellia* and *Methylophaga*, with relative abundances differing among the different treatments (Fig, S2). For the 9°C treatments, the photoperiod did not strongly affect the community composition. Here, *Colwellia* and *Methylophaga* occurred roughly in a similar relative distribution. However, at 13.5°C, *Colwellia* dominated the 16:8 h treatment, whereas at 24:0 h, *Methylophaga* dominated. In addition, smaller contributions to the respective bacterial community, including *Glaciecola*, *Sulfitobacter*, *Pseudomonas*, and *Polaribacter*, were absent in the 13.5°C 24:0 h treatment.

### Extracellular DOC concentrations and net cell-specific DOC accumulation rates

**Fig 2:**
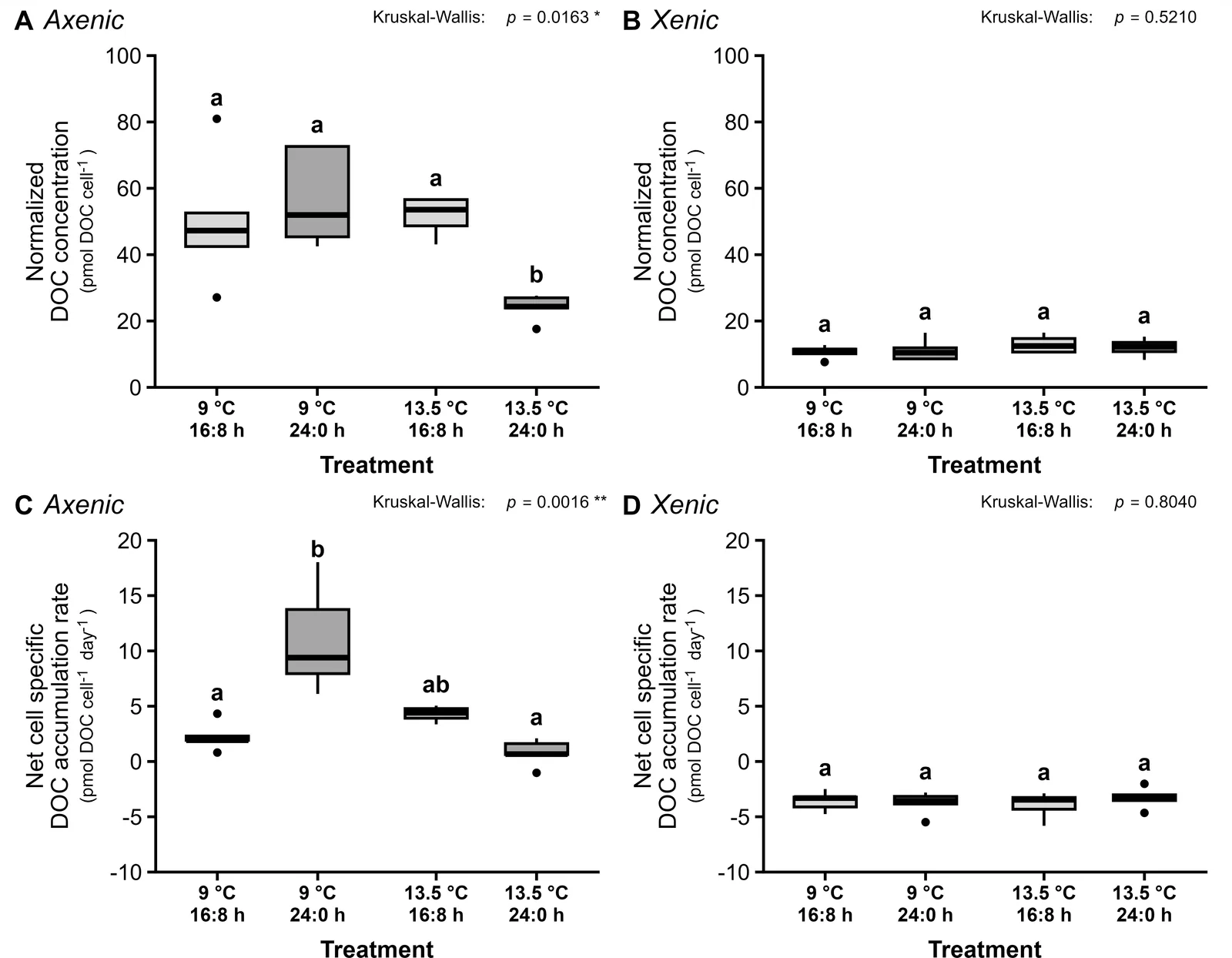
Box plots displaying DOC concentration normalized to algal cell density and net cell-specific DOC accumulation rate in filtrates of (**A, C**) axenic and (**B, D**) xenic cultures of *T. gravida* under different temperatures (9°C, 13.5°C) and photoperiods (16:8 h, 24:0 h). Each treatment consisted of five replicates with the exception of axenic cultures at 13.5°C and 24:0 h (*n* = 4). Treatments with different lowercase letters differed significantly (*P* < 0.05).

Extracellular DOC concentrations normalized to cell density were significantly higher in axenic cultures than in xenic cultures across all treatments (*P* < 0.05, Fig. 2; Table S4). The same pattern was observed for the original (non-normalized) DOC concentrations (Table S2). Relative to the axenic treatments, xenic cultures showed 36–72% lower original (non-normalized) DOC concentration and 50–80% lower cell-normalized DOC concentration.

Treatment-dependent differences in extracellular DOC concentrations could be observed only in axenic cultures. Here, the 13.5°C 24:0 h treatments exhibited significantly lower cell-normalized DOC than the other axenic treatments (52–58%; *P* < 0.05, Fig. 2A). In contrast, DOC concentrations did not differ significantly among xenic treatments (*P* = 0.521, Fig. 2B).

Similarly, net cell-specific DOC accumulation rates were significantly higher in axenic than in xenic treatments across all conditions (*P* < 0.05, Fig. 2C and D). Axenic treatments cultivated at 9°C 24:0 h were significantly higher than those at 9°C 16:8 h and 13.5°C 24:0 h. Similar to extracellular normalized DOC concentrations in xenic cultures, net accumulation rates did not differ among xenic treatments (*P* = 0.804, Fig. 2D).

### FT-ICR-MS total ion chromatogram (TIC)

**Fig 3:**
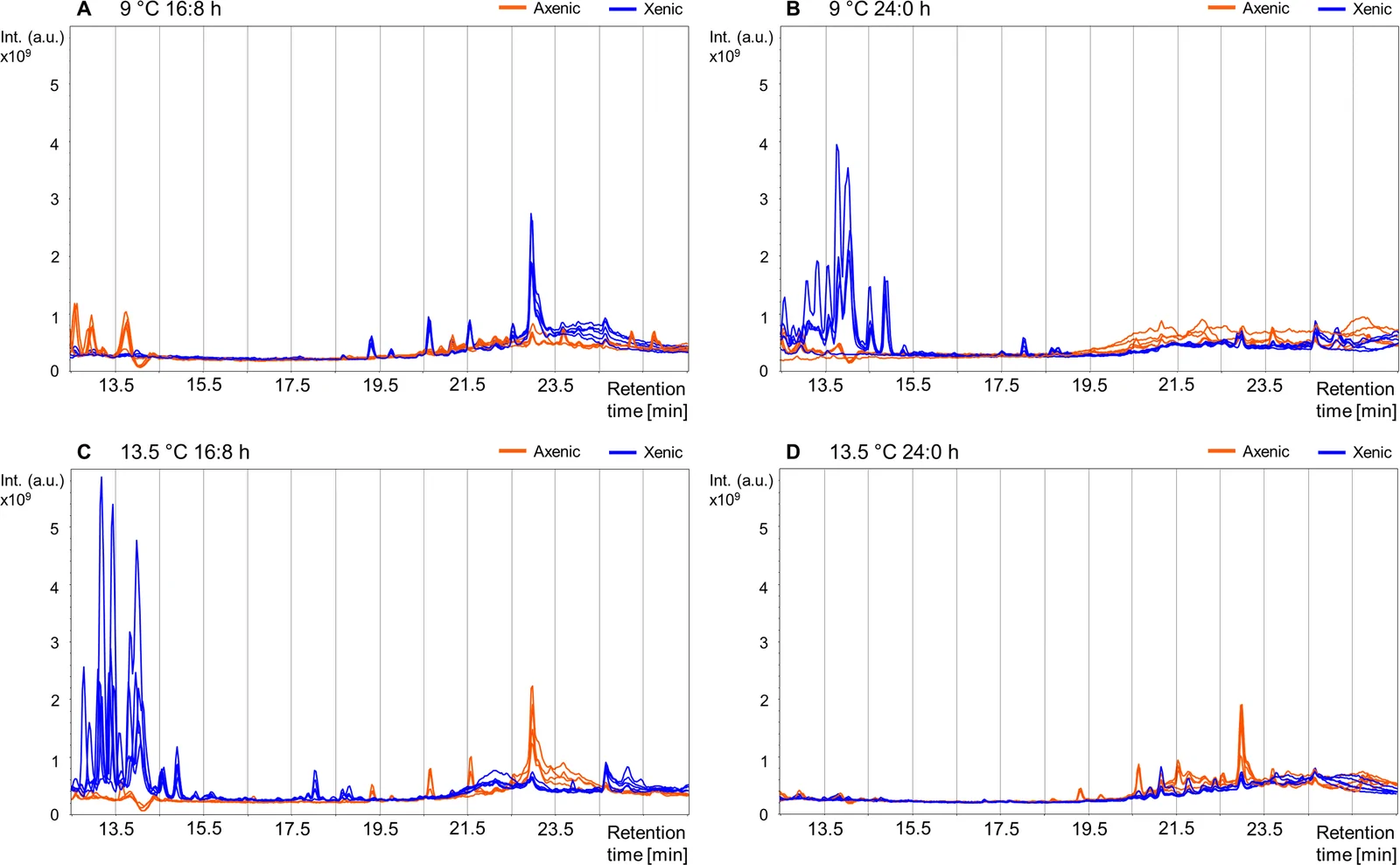
Total ion chromatograms (TICs) of filtrates of axenic (orange) and xenic (blue) cultures of *T. gravida* grown under different temperature and photoperiod conditions: 9°C 16:8 h (**A**), 9°C 24:0 h (**B**), 13.5°C 16:8 h (**C**), and 13.5°C 24:0 h (**D**). Each treatment consisted of five replicates with the exception of axenic cultures at 13.5°C and 16:8 h (*n* = 4). Mass spectrometric acquisition started at a retention time of 10 min to exclude the salt fraction (53). For subsequent molecular formula assignment, scans between 12.5 and 26.5 min were averaged into 1-min bins (vertical lines).

The TIC shows the distribution of ionizable DOM along the polarity gradient (retention time). In contrast to native marine DOM (53), our experimental samples showed several well-defined chromatographic peaks with peak widths comparable to those typically observed for individual molecules (< 0.5 min). Axenic and xenic cultures strongly differed in signal intensity and peak distribution across all experimental conditions (Fig. 3).

In all chromatograms, only a few peaks were detected between approximately 15 and 18 min, resulting in a low-intensity retention time range that separated a cluster of more polar peaks eluting early at 12.5–15 min from later-eluting and less polar peaks after 18 min.

At 9°C 16:8 h, axenic TIC profiles were dominated by an early-eluting cluster between 12.5 and 14 min (Fig. 3A). In contrast, axenic TICs in the other treatment were dominated by later-eluting signals after approximately 19 min. Under 13.5°C 16:8 h and 13.5°C 24:0 h conditions, axenic cultures additionally displayed a pronounced peak between 22.5 and 23.5 min (Fig. 3C and D).

In xenic cultures under 9°C 16:8 h conditions, TICs were dominated by late-eluting peaks between 19 and 24 min (Fig. 3A). At 9°C 24:0 h and 13.5°C 16:8 h, TICs in xenic cultures displayed an intense cluster of peaks between 12.5 and 15 min. Compared to the other xenic treatments, TICs of the xenic 13.5°C 24:0 h treatment showed the overall lowest signal intensities (Fig. 3D).

### Chemodiversity

**Fig 4:**
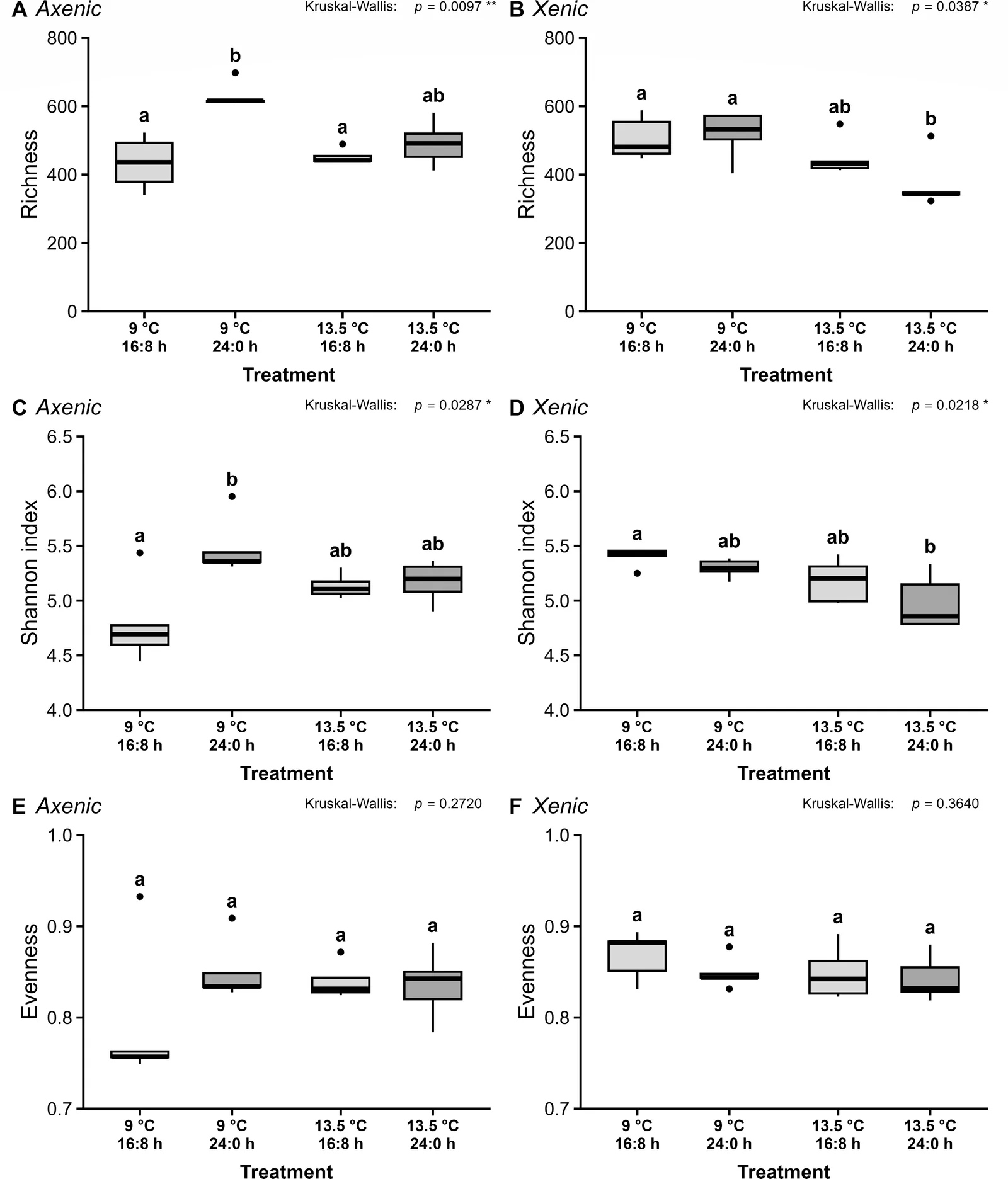
Chemodiversity parameters in filtrates for axenic and xenic *T. gravida* cultures. Richness (number of molecular formulas summed over retention time) (**A** and **B**), Shannon diversity (**C** and **D**), and evenness (**E** and **F**) under different temperature (9°C, 13.5°C) and photoperiod (16:8 h, 24:0 h) conditions. Each treatment consisted of five replicates with the exception of axenic cultures at 13.5°C and 16:8 h (*n* = 4). Treatments with different lowercase letters differed significantly (*P* < 0.05).

To assess the chemodiversity of extracellular DOM, molecular richness, the Shannon diversity, and Pielou’s evenness indices were calculated based on the peak intensities of all detected molecular formulas. Axenic cultures at 9°C at 24:0 h displayed significantly higher values for richness and Shannon diversity (45% and 14%, respectively; *P* < 0.05; Fig. 4A and C) compared to the 16:8 h light treatment. In addition, richness was significantly higher than at 13.5°C 16:8 h (39%, *P* < 0.05, Fig. 4A). In contrast, in axenic 13.5°C cultures, no significant differences could be observed between photoperiods with respect to all diversity measures. Moreover, in xenic cultures, the 13.5°C 24:0 h treatment showed significantly lower richness compared to the 9°C treatments (26% for 16:8 h and 28% for 24:0 h, *P* < 0.05, Fig. 4B), and the diversity was significantly lower than in the 9°C 16:8 h treatment (8%, *P* < 0.05, Fig. 4D).

The comparison between axenic and xenic cultures showed significantly higher richness in axenic cultures at 9°C 24:0 h (22%, *P* < 0.05, Table S4), whereas no significant differences in the Shannon index occurred between axenic and xenic cultures (*P* > 0.05, Table S4). No significant differences in the Pielou’s evenness index were observed within axenic and xenic cultures (Fig. 4E and F).

### Nominal oxidation state of carbon (NOSC), hydrogen, and oxygen saturation

**Fig 5:**
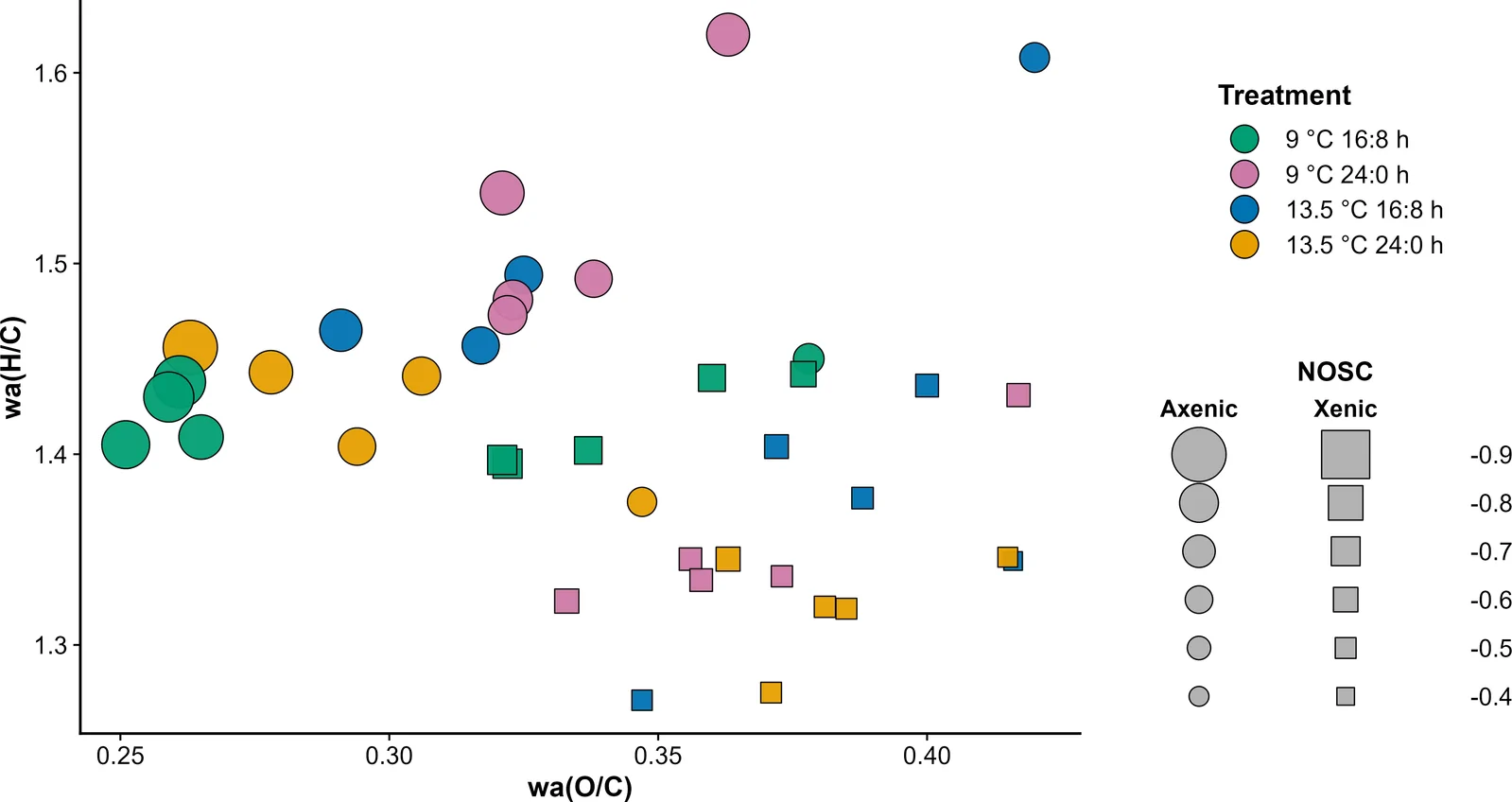
Average molecular oxygen to carbon versus hydrogen to carbon ratios (van Krevelen diagram) of filtrates of axenic (circles) and xenic (squares) cultures of *T. gravida* under different temperature and photoperiod conditions: 9°C 16:8 h (green), 9°C 24:0 h (pink), 13.5°C 16:8 h (blue), and 13.5°C 24:0 h (yellow). Each treatment consisted of five replicates with the exception of axenic cultures at 13.5°C and 16:8 h (*n* = 4). All values represent the sample-averages for the intensity-weighted average atomic oxygen to carbon (wa(O/C)) and hydrogen to carbon ratio (wa(H/C)) for the detected molecular formulas. Dot sizes represent the average nominal oxidation state of carbon (NOSC) for a sample. The more oxidized the carbon in the sample, the smaller the dot size.

NOSC represents the average oxidation state of carbon atoms in a molecule. In the experiment, NOSC ranged from −0.90 to −0.43 and was less negative in xenic than in axenic cultures (30%; *P* < 0.05, Fig. 5), with significant differences between axenic and xenic cultures across all treatments (*P* < 0.05), while no significant differences were found within axenic cultures (Table 1). In xenic cultures, NOSC in both 13.5°C treatments were significantly higher compared to the 9°C 16:8 h treatment (Table 1; 23% for 13.5°C 16:8 h and 24% for 13.5°C 24:0 h; *P* < 0.05).

**TABLE 1:** *P* values of Kruskal–Wallis test and Benjamini–Hochberg-corrected significance levels^*a*^

Variable	Axenic					Xenic				
	Kruskal–Wallis	9°C 16:8 h	9°C 24:0 h	13.5°C 16:8 h	13.5°C 24:0 h	Kruskal–Wallis	9°C 16:8 h	9°C 24:0 h	13.5°C 16:8 h	13.5°C 24:0 h
H/C	0.003*	a	b	b	a	0.042*	a	ab	ab	b
O/C	0.115	ns	ns	ns	ns	0.07	ns	ns	ns	ns
NOSC	0.225	ns	ns	ns	ns	0.007*	a	ab	b	b

^*a*^ Shown are results from Dunn’s *post hoc* test for axenic and xenic cultures of *T. gravida* under different temperature (9°C, 13.5°C) and photoperiod (16:8 h, 24:0 h) conditions. Each treatment consisted of five replicates with the exception of axenic cultures at 13.5°C and 16:8 h (*n* = 4). Axenic and xenic treatments were tested separately. Asterisks indicate *P* values < 0.05. Treatments with different lowercase letters differed significantly.

For the wa(H/C) ratio in axenic cultures, values at 9°C 24:0 h and 13.5°C 16:8 h were significantly higher than at 9°C 16:8 h (7% and 6%, respectively; *P* < 0.05), whereas no significant difference was detected for the wa(O/C) ratio within axenic cultures (Table 1). The xenic 9°C 16:8 h treatment was significantly higher than 13.5°C 24:0 h for the wa(H/C) ratio (7%; *P* < 0.05), whereas no significant treatment effect was detected for the wa(O/C) ratio in xenic cultures (Table 1).

The comparison between axenic and xenic cultures within the same treatment showed significantly lower wa(H/C) values in xenic cultures at 9°C 24:0 h (11%), 13.5°C 16:8 h (9%), and 13.5°C 24:0 h (7%; *P* < 0.05, Table S4), whereas no significant difference was detected at 9°C 16:8 h (*P* = 0.42). The wa(O/C) ratios were significantly higher in xenic cultures at 13.5°C 24:0 h than in the same axenic cultures (29%; *P* < 0.05, Table S4), while differences in wa(O/C) in the remaining treatments were not significant (*P* > 0.05).

### Degree of DOM unsaturation

**Fig 6:**
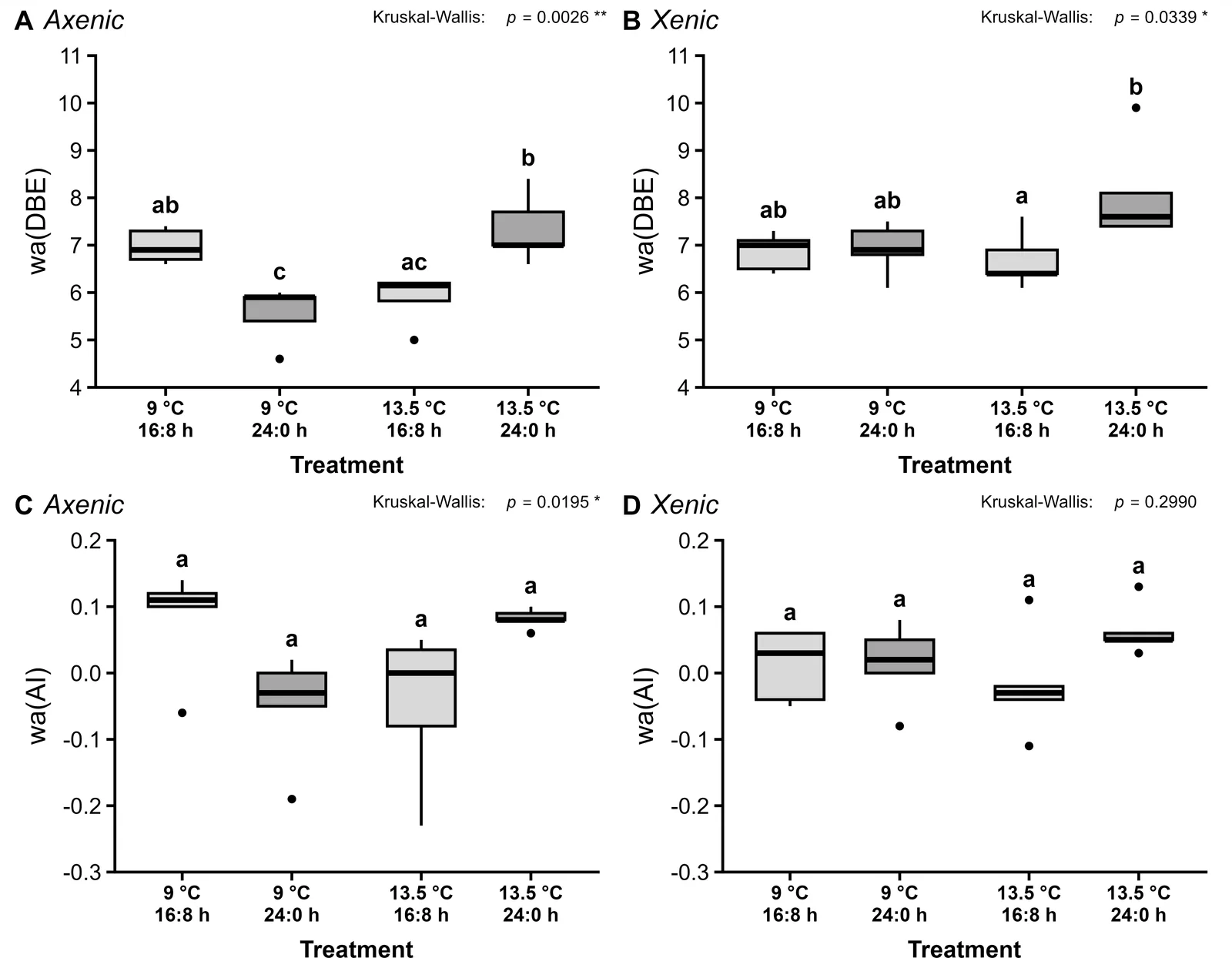
Weighted average (wa) double bond equivalents (DBE) and aromaticity index (AI) of axenic (**A, C**) and xenic (**B, D**) cultures of *T. gravida* under different temperature (9°C, 13.5°C) and photoperiod (16:8 h, 24:0 h) conditions. Each treatment consisted of five replicates with the exception of axenic cultures at 13.5°C and 16:8 h (*n* = 4). Treatments with different lowercase letters differ significantly (*P* < 0.05).

Structural features such as the degree of unsaturation correlate with the bioavailability of DOM (85, 86). In axenic cultures, wa(DBE) was significantly lower at 9°C 24:0 h than at 9°C 16:8 h and 13.5°C 24:0 h (20% and 24%, respectively; Fig. 6A; *P* < 0.05). In xenic cultures, wa(DBE) was significantly higher at 13.5°C 24:0 h than at 13.5°C 16:8 h (21%; Fig. 6B; *P* < 0.05). For wa(AI), a global treatment effect was detected in axenic cultures (Kruskal–Wallis; *P* < 0.05), but no other pairwise test was significant. In addition, no significant difference could be observed in xenic cultures regarding wa(AI), although a pattern across all experimental conditions similar to that of wa(DBE) occurred (Fig. 6D).

Significantly higher values for wa(DBE) (24%) in xenic cultures than in axenic cultures were present at 9°C 24:0 h (*P* < 0.05, Table S4), whereas wa(AI) did not differ significantly between axenic and xenic treatments (*P* > 0.05, Table S4).

## DISCUSSION

The comparison of axenic and xenic *T. gravida* cultures helps to distinguish purely physiological responses under axenic conditions from bacteria-associated net DOM processes in xenic cultures, as well as to assess their impact on extracellular DOM quantity and quality. To provoke a detectable physiological temperature response of the cold-adapted Arctic species *T. gravida*, it was necessary to set the experimental temperatures to values outside the natural range of sea-surface temperatures in the Arctic Ocean (ca. −0.9°C to 1.6°C, depending on the season) (87). Instead, we chose two temperatures based on pre-determined thermal performance curves of *T. gravida* (54) rather than to assess a continuous temperature-response gradient. Optimal performance temperatures usually lie several degrees above average environmental temperatures (88). 9°C was selected as the low temperature in our experiment, which falls within the high-performance range of *T. gravida*. For the high-temperature treatments, 13.5°C was selected, a value that was beyond the thermal optimum of the performance curve and expected to create a physiological stress response. This experiment was designed to compare DOM quantity and quality under two biologically defined temperatures. Since it cannot be distinguished whether the observed differences in extracellular DOC concentration and DOM composition originate from bacterial consumption or bacterial production, we interpret the differences between axenic and xenic cultures as “bacterial net effects” on extracellular DOM.

### Does the 13.5°C 24:0 h treatment result in carbon reallocation into biomass?

The reallocation of energy and carbon in response to varying environmental conditions is a common response by phytoplankton to optimize growth (89 and references therein). In the 13.5°C, 24:0 h treatment, axenic cultures showed the highest growth (Fig. 1A), contrary to the results from a previous study (40), the lowest normalized extracellular DOC concentrations (Fig. 2A), and the net cell-specific DOC accumulation rate (Fig. 2C). This potentially reflects that fixed carbon was allocated into cell material rather than accumulating as extracellular DOC.

Similar net cell-specific DOC accumulation rates under axenic 9°C 16:8 h and 13.5°C 24:0 h conditions coincided with contrasting growth dynamics: 9°C 16:8 h required 12 days to reach harvest density and showed the lowest specific growth rate, whereas 13.5°C 24:0 h required only 4 days and showed the highest growth rate (Table S1). Because this metric represents net change, it cannot resolve whether carbon was re-assimilated or partitioned into cellular or particulate pools.

Mechanistically, elevated temperatures accelerate metabolic processes (90–92), and in combination with a continuous supply of light, these favor rapid incorporation of fixed carbon into biomass. Consequently, this may have resulted in a smaller net fraction of fixed carbon remaining as dissolved exudates, so that the low net DOC accumulation at 13.5°C 24:0 h, compared to 9°C and 16:8 h, indicated the coupling of carbon assimilation to growth, potentially reducing the need for carbon diversion to dissolved exudates. This is supported by previous findings for the same experiment demonstrating that with increasing photoperiod and temperature, *T. gravida* reduced the expression of various genes for transport and alginic acid biosynthesis (61). Since dissolved carbohydrates can represent a fraction of marine DOM (93, 94), a reduced alginic acid production potentially contributes to lower extracellular DOC concentrations. As described by the "integrated growth environment" concept, environmental conditions jointly influence growth dynamics of phytoplankton, resulting in different fates of photosynthetic products, enabling partitioning of carbon pathways as acclimation strategies (95). Here, photosynthetically acquired resources are allocated among all cellular processes, depending on temperature, light conditions, and nutrient availability. In our study, the combination of elevated temperature and prolonged photoperiods may have favored the coupling between photosynthetic carbon and cellular growth.

The high specific growth rates and low extracellular DOC concentrations and net cell-specific DOC accumulation rates can also be interpreted in the framework of the overflow and passive diffusion hypotheses. Regarding the passive diffusion model (i.e., the maintenance of a DOC gradient across the cell membrane), low extracellular DOC concentrations and net cell-specific DOC accumulation rates might represent passive diffusion as a background process (47). Regarding the overflow hypothesis (i.e., the extracellular release of excess photosynthate which cannot be incorporated into the cell), elevated temperatures could be expected to yield higher extracellular DOC concentrations for the cold-adapted species *T. gravida* due to increased photosynthetic activity. For instance, it has been demonstrated that warming can increase DOC production in *Phaeodactylum tricornutum* (96). In a similar manner, prolonged photoperiods could be expected to result in higher extracellular DOC concentrations due to extended periods for carbon fixation. Few studies demonstrated that higher irradiances may impact DOC release in phytoplankton cultures (97–99). Thus, our results do not indicate an overflow in the 13.5°C 24:0 h treatment, possibly due to altered carbon partitioning into cell material, and add specificity within the overflow and passive diffusion framework by indicating that varying environmental conditions may not only affect extracellular release but also determine to which extent DOC accumulates in the extracellular environment (52% in the 13.5°C 24:0 h treatment compared to 9°C 16:8 h).

All of the above observations were only possible under axenic conditions and none of the reported differences were observed in the xenic treatments (Fig. 2B and D; Table S2; see discussion below).

### Axenic cultures: longer photoperiods at optimum growth temperature change the exudate composition

Several studies have characterized the composition of DOM derived from diatoms (60, 100–102). However, there is a knowledge gap addressing the composition of exudates in response to varying environmental conditions, particularly diversity and bioavailability metrics. These metrics are important for a better mechanistic understanding of the bacterial degradation of algal-derived DOM in the changing Arctic Ocean. Since the bacterial turnover is fast in the natural environment, axenic cultures represent a unique opportunity to study the chemical composition of the original substrate source and how it relates to temperature and light.

It needs to be considered that algal exudation and photo alteration caused by prolonged photoperiods cannot be distinguished under the applied experimental conditions. This is especially relevant for the 24:0 h photoperiod treatments since prolonged photoperiods may affect the DOM composition independent from biological processes. DOM composition in the natural environment can be affected by both microbial and abiotic transformations (103, 104). However, photochemical alteration processes alone are unlikely to be connected to treatment-dependent DOM patterns. Both temperature regimes included a 24:0 h photoperiod treatment, and the observed differences made at 9°C could not be reproduced at 13.5°C treatments. Hence, it is possible that the effects of prolonged photoperiods were modulated by temperature-dependent algal physiology. Nevertheless, the DOM patterns observed under the 24:0 h photoperiod treatments should be interpreted as net treatment differences resulting from the DOC release of *T. gravida* as well as potential photochemical alteration and cannot be attributed exclusively to biological processes.

The number of detected molecular formulas (richness) and the chemodiversity were highest in axenic treatments cultured at 9°C and 24:0 h (Fig. 4A and C), coherent with the highest net cell-specific DOC accumulation rates (Fig. 2C). Evenness (Fig. 4E) was higher at 9°C 24:0 h compared to 9°C 16:8 h, even though no statistical significance could be observed. This suggests that extreme light regimes at optimal growth temperatures were likely responsible for the net increase of chemical diversity exudated by *T. gravida*. At constant illumination, exudates contained more molecular formulas and showed a more even abundance distribution compared to 16:8 h. The net effect of constant illumination on chemodiversity could be mechanistically explained by the prolonged passive export of intracellular metabolites. Since diatoms can downregulate their metabolic and transcriptomic activity during dark cycles (105), long photoperiods could result in a continuous production of additional metabolites that are exuded into the medium.

The exudate composition under axenic conditions and constant light at optimal growth temperature reflected a DOM pool of higher bioavailability. Compared to axenic 9°C 16:8 h, molecular formulas were more saturated, as indicated by higher H/C ratios (Fig. 5; Table 1, *P* < 0.05) and lower DBE (Fig. 6A, *P* < 0.05). This is in agreement with several previous studies showing that molecular formulas with high H/C ratios are often preferentially utilized, whereas aromatic, low H/C ratio formulas tend to persist in aquatic environments (106–109). O/C ratios in axenic treatments were higher and NOSC was lower in 9°C 24:0 h treatments than in the 9°C 16:8 h treatments (Fig. 5), although this tendency was not statistically significant (Table 1). The NOSC quantifies the average oxidation state of carbon independent from the compounds’ chemical structures (110) and thus serves as an indicator for the bioenergetic potential of DOM, with high NOSC values making the oxidation of organic matter thermodynamically more favorable (72, 111). The O/C ratio is closely related to NOSC and reflects the relative abundance of oxygen-containing functional groups such as carboxyl and hydroxyl moieties (112, 113). While an increasing O/C ratio reflects a higher degree of oxidation, the link to DOM degradability is yet inconclusive. It was summarized that the literature reports negative (106), positive (114), or no correlation between O/C ratios (115) and bioavailability/biodegradation in different DOM settings (116). Consequently, the O/C ratio alone should not be applied as a proxy for bioavailability and/or reactivity. High wa(H/C) and moderate wa(O/C) ratios in the axenic 9°C 24:0 h treatments of our study locate most formulas in the unsaturated aliphatic region of the van Krevelen space (117) (Fig. S1). Upon various environmental perturbations, *T. pseudonana* have been shown to reallocate carbon through primary metabolism and accumulate tricarboxylic acid cycle (TCA) intermediates and related LMW compounds, including various small organic acids (118, 119). Further studies of *T. pseudonana* demonstrate that a subset of such metabolites is released into the dissolved phase and contributes to the diatom’s exometabolome (101, 120, 121). The substrate heterogeneity (as indicated by higher molecular formula diversity) in cultures of 9°C 24:0 h might promote the partitioning of certain bacterial taxa in the phycosphere, favoring those capable of targeting specific compounds (122–125). It has been shown that the bioavailability of organic exudates of *Chaetoceros neogracilis* and *Fragilariopsis cylindrus* is taxon-specific, ultimately influencing bacterial community composition (126).

However, the net photoperiod effect on extracellular DOM composition, i.e., higher chemodiversity, and wa(H/C) together with lower wa(DBE) could not be observed under 13.5°C (Fig. 4A, C and D; Fig. 5 and 6A). As discussed for the cell-normalized extracellular DOC concentrations, this may reflect a preferential carbon allocation to growth rather than accumulation in the dissolved phase.

In comparison to the overflow hypothesis, our data suggest that the excess carbon does not result in a quantitative overflow that accumulates extracellularly, but rather in a diversification of the extracellular DOM pool. This demonstrates that extracellular release should be studied not exclusively on a quantitative, but also on a qualitative level.

### Molecular composition in the xenic 13.5°C 24:0 h treatments indicates enhanced net DOM processing

Many previous studies have shown that algal exudates are largely bioavailable and metabolized by bacteria (16). The microbial turnover produces a small fraction of non-labile compounds, which act as precursors for recalcitrant DOC (15). We aimed to understand how the 13.5°C 24:0 h condition affected extracellular DOC concentration and composition and how compositional changes may affect its bioavailability and contribution to the efficacy of the MCP. As discussed before, it needs to be considered that the observed changes were a net result of DOC release from *T. gravida*, bacterial processing, and potential photochemical alteration.

In accordance with the bacteria-associated net processing of diatom-derived DOC, all cell-normalized extracellular DOC concentrations were very similar and lower compared to the axenic treatments (Fig. 2). Inorganic nutrient limitation can be ruled out as an explanation because nutrients were consistently high throughout the experiment (Table S3). All net cell-specific DOC accumulation rates were also very similar and negative, indicating that bacteria-associated net processing mainly contributed to the ultimate concentration of background DOC, independent of temperature, photo-effects, or community composition. However, the chemical characteristics of the xenic treatments displayed significant differences, such as lower chemical richness and diversity at 13.5°C 24:0 h compared to 9°C 16:8 h (Fig. 4B and D). Because evenness did not differ between these treatments (Fig. 4F), the decrease in Shannon diversity, which is calculated from richness and evenness, is primarily driven by the lower number of detected molecular formulas rather than a shift in their relative abundance. This observation is corroborated by a generally lower signal intensity in the chromatograms (TIC) of the xenic cultures at 13.5°C 24:0 h (Fig. 3D). However, it is also possible that the reduced signal may also indicate a lower abundance of ionizable DOM thus affecting richness and chemodiversity.

As shown above, axenic 13.5°C 24:0 h cultures yielded less extracellular DOC. In the presence of bacteria in the respective xenic cultures, we observed a significantly lower chemical richness and diversity. It is possible that less extracellular DOC provided by *T. gravida* constrained the substrate pool available for the DOM transformation processes associated with the free-living bacteria, i.e., in assemblages dominated by *Methylophaga* and *Colwellia*, with smaller contributions of *Sulfitobacter* and *Glaciecola* (Fig. S2). Less phytoplankton DOC could alter interkingdom exchanges in the phycosphere and putatively diminish mutualistic relationships between diatom host and bacteria (40). It has also been shown that the fraction of primary production released as DOC could be linked to bacterial cell survival (127) and structure bacterial succession during phytoplankton blooms (128). The shifts in free-living bacterial assemblages observed in our experiment, particularly regarding *Colwellia* and *Methylophaga*, suggested that the combination of temperature and light resulted in distinct community structures, potentially reflecting differences in the extracellular substrate availability generated under the respective conditions.

On a qualitative level, temperature increase and prolonged photoperiods (xenic 13.5°C 24:0 h) led to less saturated and more oxidized extracellular DOM compared to the 9°C 16:8 h treatment (Fig. 5; Table 1), and wa(DBE) at 13.5°C 24:0 h showed the highest median across all treatments. All of these observations are consistent with a higher processed state of extracellular DOM in xenic 13.5°C 24:0 h cultures (85, 129). This is in agreement with previous findings reporting accelerated respiration rates under warmer and prolonged-light conditions in Arctic plankton (e.g., 130, 131). It should be noted that incubation durations were nearly constant in xenic treatments (≤1 day difference; Table S1), making it unlikely that the observed differences were driven by longer periods of net bacterial transformation of extracellular DOM.

Higher temperature and continuous light may shift extracellular DOM towards a more processed molecular signature, but it remains to be tested if this enhances persistence and carbon sequestration.

### Xenic cultures showed more oxidized extracellular DOM and weaker treatment-specific differences than axenic cultures

Across the different temperature and light regimes, axenic cultures revealed the DOM response of *T. gravida* alone, whereas xenic cultures captured the net molecular response arising from both *T. gravida* and its associated bacteria. The comparison showed that the treatment-dependent responses observed in axenic *T. gravida* cultures were not reflected in the corresponding xenic cultures under the same environmental conditions.

Compared to axenic cultures, extracellular DOM in xenic cultures exhibited significantly lower wa(H/C), and higher wa(O/C) and NOSC values as a result of bacteria-associated net turnover. Moreover, richness was significantly lower and wa(DBE) significantly higher in xenic cultures relative to axenic cultures only at 9°C 24:0 h. Here, higher chemodiversity and more saturated DOM in axenic cultures were not reflected in xenic cultures, indicating that these photoperiod-driven effects at 9°C 24:0 h observed in axenic cultures were reduced or counterbalanced in the presence of free-living bacteria, i.e., *Colwellia-* and *Methylophaga-*dominated bacterial communities. This may contribute to the observation that bacteria can alter algal extracellular release (132, 133) and vice versa (134), highlighting how diatom-bacteria consortia can act as tightly coupled networks instead of as separate producer and consumer constituents. This observation supports the assumption that phytoplankton-bacteria interactions can affect production at a community scale (122).

In accordance with the phycosphere concept, which acts as a “marketplace” of various molecular compounds between phytoplankton and bacteria (20), metabolite exchange can restructure interactions and potentially biogeochemical outcomes (122). Several laboratory experiments have shown how bacteria influence the physiology of phytoplankton through exometabolites (123). In alignment with this concept, the comparison between axenic and xenic cultures emphasizes that, in xenic cultures, not only extracellular DOC accumulation was affected, but also net molecular DOM patterns of *T. gravida* resulting from different temperature and photoperiod conditions. Overall, extracellular DOM in xenic cultures represents a net DOM signature that is shaped by algal release, bacterial transformation, and potential bacterial release and may indicate that the bacterial presence influences the treatment-dependent extracellular DOM signatures observed in axenic *T. gravida* cultures.

In conclusion, temperature and photoperiod altered both the quantity and molecular composition of extracellular DOM released by *T. gravida*. Under axenic 13.5°C 24:0 h conditions, high growth rates coincided with low extracellular DOC accumulation, suggesting preferential carbon allocation to biomass. At 9°C, continuous light increased DOM chemodiversity and shifted molecular indicators towards a more bioavailable composition, demonstrating that changes in DOM quality can occur without corresponding changes in bulk DOC concentrations. These treatment-specific patterns were modified in xenic cultures, where bacterial processing reduced extracellular DOC and resulted in a more oxidized, less saturated DOM signature. Thus, algal carbon release and bacterial transformation constitute closely coupled processes that jointly determine extracellular carbon quantity and composition. Accounting for both DOM quantity and molecular quality is therefore essential for assessing how warming and changing light regimes may affect microbial carbon cycling in the Arctic Ocean.

Advancing knowledge about the response of microbial carbon transformations to a changing ocean is crucial for understanding and predicting the impact of climate change on the ocean carbon cycle (135, 136). Since only two temperature conditions were tested, our results should not be interpreted as a continuous thermal response curve and do not allow the evaluation of non-linear responses, thresholds, or temperature optima. Hence, future experiments should address wider temperature gradients, including more tested temperatures. To distinguish between bacterial consumption and production, these experiments should be conducted with isotope-labeled DOM. Further multifactorial experiments across species, combinations of applied environmental conditions, and growth phases are needed to fully understand how warming and varying light regimes impact carbon transformation and sequestration in the polar oceans.

## Data Availability

All data sets are archived and available from the PANGAEA data repository (137–140). Raw 16S rRNA gene amplicon sequencing data were deposited in the European Nucleotide Archive (ENA) under the accession number PRJEB115746.

## SUPPLEMENTAL MATERIAL

Supplemental material aem.01429-26-s0001.docx Fig. S1 and S2; Tables S1 to S4.

ASM does not own the copyrights to Supplemental Material that may be linked to, or accessed through, an article. The authors have granted ASM a non-exclusive, world-wide license to publish the Supplemental Material files. Please contact the corresponding author directly for reuse.
