# Supplementary material for: The diatom Thalassiosira gravida under Arctic change: algal exudates and their microbial transformation under contrasting temperatures and photoperiods
Source: Appl Environ Microbiol. 2026 Sep 4;92(10):e01429-26. doi: 10.1128/aem.01429-26 (PMC13689408; doi:10.1128/aem.01429-26)
Supplement: Supplementary file 1 [file aem.01429-26-s0001.docx]

# Supplementary Figures

**Figure S1:** van Krevelen diagrams of unique molecular formulas detected in at least three of five filtrates of axenic and xenic cultures of T. gravida. Cultivation was performed under different temperature and photoperiod conditions: 9 °C 16:8 h (a), 9 °C 24:0 h (b), 13.5 °C 16:8 h (c) and 13.5 °C 24:0 h (d). Different classes of molecular formulas are indicated by colors in the legend. “Axenic-only” plots represent formulas exclusively present in axenic cultures, “xenic-only” formulas exclusively present in xenic cultures and “shared” formulas present in both cultures for each environmental condition.

**Figure S2:** Relative abundance of free-living bacteria in xenic cultures of T. gravida under four temperature and photoperiod conditions. Taxonomic composition is shown at the genus level after stringent quality filtering. The figure was modified after (1).

# Supplementary Tables

**Table S1:** Duration of incubation (Δt, days) and cell densities at the end of the incubation of axenic and xenic cultures of T. gravida under the four experimental temperature and photoperiod conditions. Experimental cultures were inoculated at an initial cell density of 1500 cells mL⁻¹.

|  |  | **Axenic** | | | | **Xenic** | | | |
| --- | --- | --- | --- | --- | --- | --- | --- | --- | --- |
|  |  | **9 °C**  **16:8 h** | **9 °C**  **24:0 h** | **13.5 °C**  **16:8 h** | **13.5 °C**  **24:0 h** | **9 °C**  **16:8 h** | **9 °C**  **24:0 h** | **13.5 °C**  **16:8 h** | **13.5 °C**  **24:0 h** |
|  | **Δt** | **12** | **5** | **7** | **4** | **4** | **3** | **3** | **3** |
| **Replicate**  [cells mL^-1^] | **1** | 12584 | 10868 | 7384 | 13729 | 12532 | 15653 | 13417 | 17213 |
|  | **2** | 7072 | 10816 | 7696 | 10712 | 14197 | 18409 | 12220 | 14509 |
|  | **3** | 10244 | 11856 | 7488 | 12116 | 13573 | 16745 | 17161 | 16485 |
|  | **4** | 8112 | 8476 | 10244 | 13156 | 18721 | 12896 | 13885 | 14665 |
|  | **5** | 5668 | 12272 |  | 11908 | 16069 | 15965 | 13989 | 15861 |

**Table S2:** Original DOC concentrations in filtrates of axenic and xenic cultures of T. gravida (n = 5) under different temperatures (9 °C, 13.5 °C) and photoperiods (16:8 h, 24:0 h). Values represent the mean ± standard deviation.

|  | **Axenic** | | | | **Xenic** | | | |
| --- | --- | --- | --- | --- | --- | --- | --- | --- |
|  | **9 °C**  **16:8 h** | **9 °C**  **24:0 h** | **13.5 °C**  **16:8 h** | **13.5 °C**  **24:0 h** | **9 °C**  **16:8 h** | **9 °C**  **24:0 h** | **13.5 °C**  **16:8 h** | **13.5 °C**  **24:0 h** |
| **c(DOC)**  [µmol L^-1^] | 398 ± 48 | 614 ± 166 | 434 ±  42 | 294 ± 34 | 157 ± 21 | 173 ± 31 | 181 ± 25 | 187 ± 30 |

**Table S3:** Nutrient concentrations in filtrates of axenic and xenic cultures of T. gravida (n = 5) under different temperatures (9 °C, 13.5 °C) and photoperiods (16:8 h, 24:0 h). Values represent the mean ± standard deviation.

|  | **Axenic** | | | | **Xenic** | | | |
| --- | --- | --- | --- | --- | --- | --- | --- | --- |
|  | **9 °C**  **16:8 h** | **9 °C**  **24:0 h** | **13.5 °C**  **16:8 h** | **13.5 °C**  **24:0 h** | **9 °C**  **16:8 h** | **9 °C**  **24:0 h** | **13.5 °C**  **16:8 h** | **13.5 °C**  **24:0 h** |
| Nitrate  [µmol L^-1^] | 440.7 ± 32.2 | 448.5 ± 11.8 | 456.5 ± 29.6 | 479.9 ± 11.8 | 409.5 ± 14.4 | 430.7 ± 15.4 | 426.2 ± 13.3 | 440.1 ± 18.5 |
| Nitrite  [µmol L^-1^] | 0.8 ± 0.1 | 0.6 ± 0.0 | 0.9 ± 0.2 | 0.4 ± 0.0 | 0.4 ± 0.1 | 0.2 ± 0.1 | 0.1 ± 0.0 | 0.3 ± 0.0 |
| Phosphate  [µmol L^-1^] | 22.6 ± 3.1 | 21.0 ± 1.8 | 22.2 ± 4.0 | 24.8 ± 1.5 | 19.0 ± 1.7 | 24.2 ± 1.5 | 23.4 ± 1.5 | 20.8 ± 2.2 |
| Silicate  [µmol L^-1^] | 36.7 ± 11.4 | 16.8 ± 4.2 | 5.6 ± 4.4 | 12.8 ± 4.1 | 5.9 ± 0.1 | 5.3 ± 1.4 | 10.6 ± 2.3 | 7.1 ± 0.4 |

**Table S4:** Exact p-values resulting from a two-sided Mann-Whitney-U-test comparing axenic and xenic cultures of T. gravida within each treatment. Cultures were cultivated under different temperatures (9 °C, 13.5 °C) and photoperiods (16:8 h, 24:0 h). Cliff’s δ was calculated as an effect-size measure, with positive values indicating higher values in axenic cultures and negative values indicating higher values in xenic cultures. DOC: dissolved organic carbon, wa: peak-magnitude weighted average, AI: aromaticity index, DBE: double bond equivalents.

|  | **Treatment** | | | | | | | |
| --- | --- | --- | --- | --- | --- | --- | --- | --- |
|  | **9 °C 16:8 h** | | **9 °C24:0 h** | | **13.5 °C 16:8 h** | | **13.5 °C24:0 h** | |
|  | ***p*** | **Cliff’s δ** | ***p*** | **Cliff’s δ** | ***p*** | **Cliff’s δ** | ***p*** | **Cliff’s δ** |
| **Specific growth rate** | 0.008 | -1 | 0.008 | -1 | 0.016 | -1 | 0.008 | -1 |
| **Normalized DOC conc.** | 0.008 | 1 | 0.008 | 1 | 0.016 | 1 | 0.008 | 1 |
| **Net cell specific DOC acc. rate** | 0.008 | 1 | 0.008 | 1 | 0.016 | 1 | 0.008 | 1 |
| **Richness** | 0.222 | -0.52 | 0.008 | 1 | 0.214 | 0.55 | 0.056 | 0.76 |
| **Shannon diversity** | 0.056 | -0.76 | 0.222 | 0.52 | 0.905 | -0.1 | 0.222 | 0.52 |
| **Evenness** | 0.151 | -0.6 | 0.841 | -0.12 | 0.905 | -0.1 | 1 | -0.04 |
| **wa(H/C)** | 0.238 | 0.44 | 0.008 | 1 | 0.016 | 1 | 0.008 | 1 |
| **wa(O/C)** | 0.079 | -0.68 | 0.095 | -0.68 | 0.206 | -0.55 | 0.008 | -1 |
| **NOSC** | 0.048 | -0.76 | 0.008 | -1 | 0.016 | -1 | 0.008 | -1 |
| **wa(AI)** | 0.127 | 0.6 | 0.198 | -0.52 | 0.952 | 0.05 | 0.175 | 0.56 |
| **wa(DBE)** | 0.595 | 0.24 | 0.008 | -1 | 0.087 | -0.75 | 0.286 | -0.44 |

**References**

1. Giesler, J. (2024). The diatom holobiont in a changing Arctic Ocean. University of Bremen, Bremen. doi: 10.26092/elib/3686
